# Tomographic assessment of palatal suture opening pattern and pterygopalatine suture disarticulation in the axial plane after midfacial skeletal expansion

**DOI:** 10.1186/s40510-020-00321-9

**Published:** 2020-07-20

**Authors:** Ozge Colak, Ney Alberto Paredes, Islam Elkenawy, Martha Torres, Joseph Bui, Sara Jahangiri, Won Moon

**Affiliations:** School of Dentistry, Center for Health Science, University of California at Los Angeles, Room 63-082 CHS, 10833 Le Conte Ave, Box 951668, Los Angeles, CA 90095-1668 USA

**Keywords:** Maxillary expansion, Midpalatal suture, Midfacial skeletal expander (MSE), Cone beam computed tomography (CBCT)

## Abstract

**Objective:**

The purpose of this study was to assess the palatal suture opening and the pterygopalatine suture disarticulation pattern in the tomographic axial plane after treatment with midfacial skeletal expander (MSE).

**Materials and methods:**

Pre- and post-expansion CBCT records of 50 subjects (20 males, 30 females, mean age 18 ± 3 years) who were treated with MSE (Biomaterials Korea, Seoul, Korea) appliance were superimposed and compared using OnDemand software. Reference planes were identified and the angulation of the midpalatal suture opening after expansion was calculated as well as the frequency of the pterygopalatine suture split.

**Results:**

After MSE treatment, the mean palatal suture opening angle (SOA) was 0.57°. (− 0.8° to 1.3°). There was no significant difference between males and females in terms of the palatal suture opening pattern (*P* > 0.05). Only 3 out of 50 (6%) subjects presented SOA above 1 degree. Also, 3 out of 50 (6%) patients presented a negative SOA value. With regard to the pterygopalatine suture split, 84 sutures out of 100 (84%) presented openings between the medial and lateral pterygoid plates on both right and left sides. Partial split was detected with 8 patients (5 females, 3 males). Five patients had split only in the medial pterygoid plates of both pterygomaxillary sutures, and 3 patients exhibited disarticulation on the right side only. No significant differences were found in the frequency of suture opening between males and females (*P* = 1.000).

**Conclusions:**

MSE appliance performed almost parallel expansion in the axial view. Remarkably, this study shows that pterygopalatine suture can be split by MSE appliance without the surgical intervention; the disarticulation of pterygopalatine suture was visible in most of the patients.

## Introduction

Maxillary transverse deficiency is a common problem in daily orthodontic practice [1, 2]. There are many treatment modalities to correct the maxillary-mandibular transverse discrepancy. Treatment options consist of orthodontic, non-surgical orthopedic and surgical correction. Orthodontists traditionally use rapid palatal expansion (RPE) to manage transverse maxillary deficiency in young patients, but interlocking of the palatal suture after puberty [3] can cause unwanted side effects with RPE treatment, such as dental tipping and alveolar bone bending, causing limited skeletal movement and poor long-term stability [4, 5]. For mature patients, the surgically assisted rapid palatal expansion (SARPE) is often applied; however, surgical morbidity should be considered. Furthermore, high cost and complex treatment process [6] are involved with SARPE. In recent years, orthodontists developed the micro-implant-assisted rapid palatal expansion (MARPE) in order to avoid the unwanted side effects and complexity, as discussed above.

With the proliferation of various MARPE designs, the midfacial skeletal expander (MSE), as particular type of MARPE, became a popular treatment option for transverse maxillary deficient patients, especially for mature patients. Among many special features, the MSE differentiates itself from other types of MARPE by its bi-cortical engagement of the four micro-implants (MI) into the palate and nasal cortical bones [5, 7], immediately lateral to midpalatal suture [8], in the posterior aspect of the maxilla between the zygomatic buttress bones. Because of this posterior force vector, the pattern of expansion for MSE is notably different from those with other expanders. RPE and MARPE generally cause a V-shaped expansion with greater opening in the anterior region [9, 10]. In contrast, more posterior expansion was observed with MSE [11, 12].

With the advancement of CBCT and the advent of novel computer software, reconstruction of skull and generation of multiplanar views allow accurate assessments of the craniofacial complex and its changes. To date, palatal suture opening pattern after tooth-borne and tooth-bone-borne RPE have been investigated using CBCT in growing patients [11, 13, 14]; nevertheless, there is little information about the pterygopalatine suture disarticulation pattern [11].

The main goal of this paper was to evaluate the pattern of midpalatal suture opening and the impact on the pterygopalatine suture, after MSE treatment.

## Material and methods

**Fig. 1:**
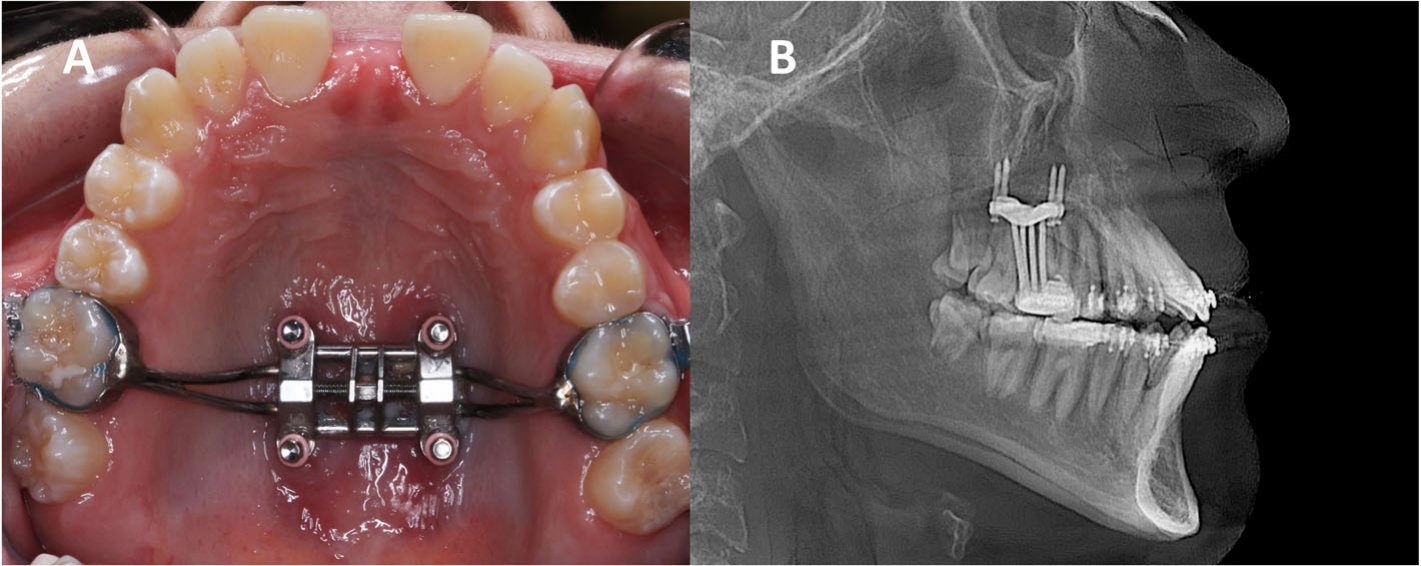
Midfacial skeletal expander (MSE). **a** Intraoral occlusal view. **b** X-ray showing bicortical engagement of MSE

Institutional review board approval (IRB number 17-000567) was granted by the University of California, Los Angeles (UCLA), to perform this retrospective study. The study included pre- and post-expansion CBCT records of 50 subjects (20 males, 30 females), who were treated with MSE (Biomaterials Korea, Seoul, Korea) appliance (Fig. 1).

**Fig. 2:**
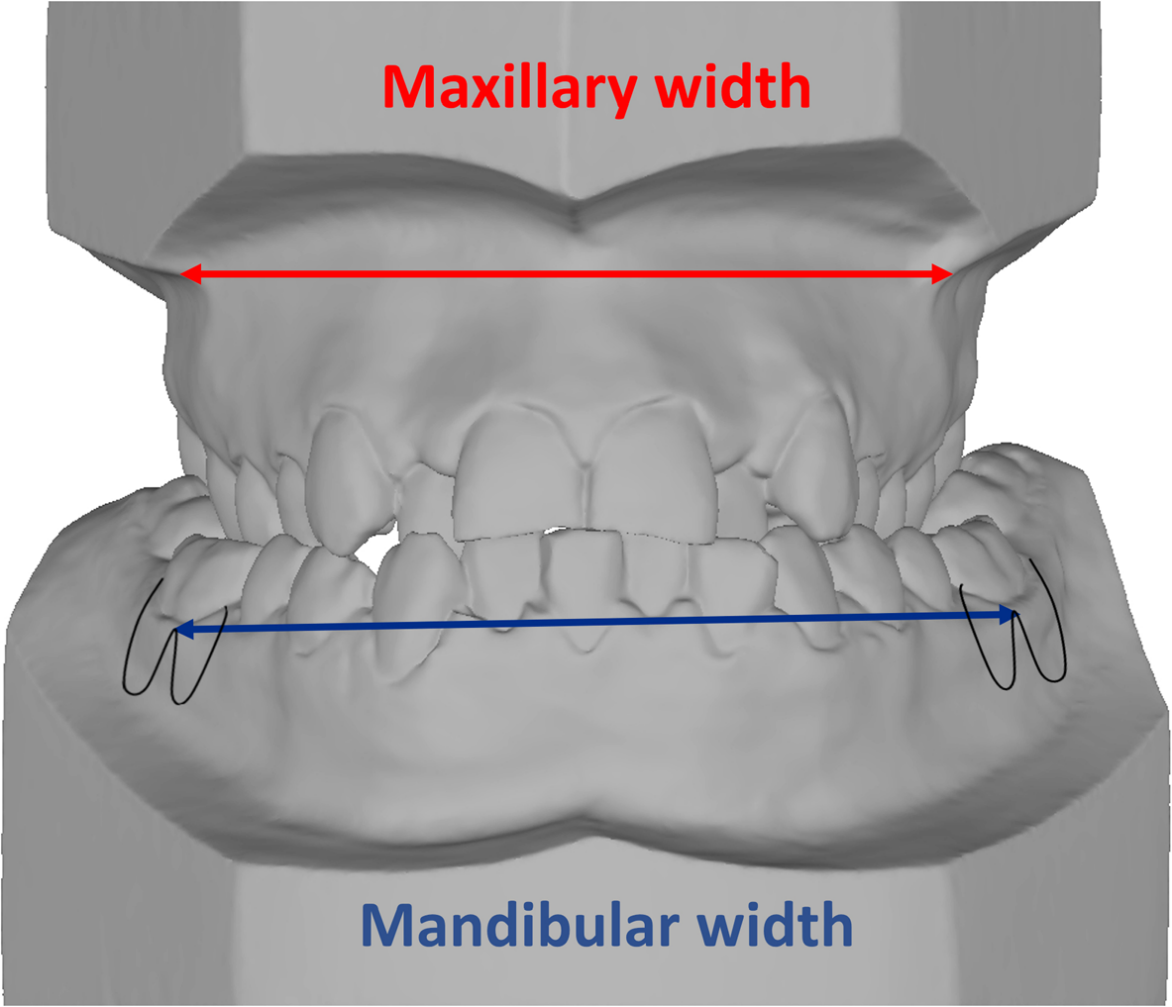
Method used to diagnose transverse maxillary skeletal deficiency. Red line: maxillary width. Blue line: mandibular width

The mean age of the subjects was 18 ± 3 years (range of 10–27 years). The selection criteria used in this retrospective study include patients diagnosed with maxillary transverse deficiency and treated at the Section of Orthodontics, UCLA School of Dentistry, under the supervision of one clinician. The transverse maxillary deficit was diagnosed by evaluating the difference between the mandibular and maxillary bone widths. The distance between the right and left gingiva tissue projected at the level of first molar’s furcation was measured to assess the mandibular bone width. The previously published WALA ridge [15] was not used due to its difficulty to locate when the buccal surface has an excessive lingual inclination with continuous slope and a prominent buccal ridge does not exist. Maxillary bone width is calculated by measuring the distance between the right and left most concave points on the maxillary vestibule, at the level of the mesiobuccal cusps of the first molars (Fig. 2). Exclusion criteria included patients with craniofacial syndromes and patients with any systemic diseases that could alter the results of treatment.

**Fig. 3:**
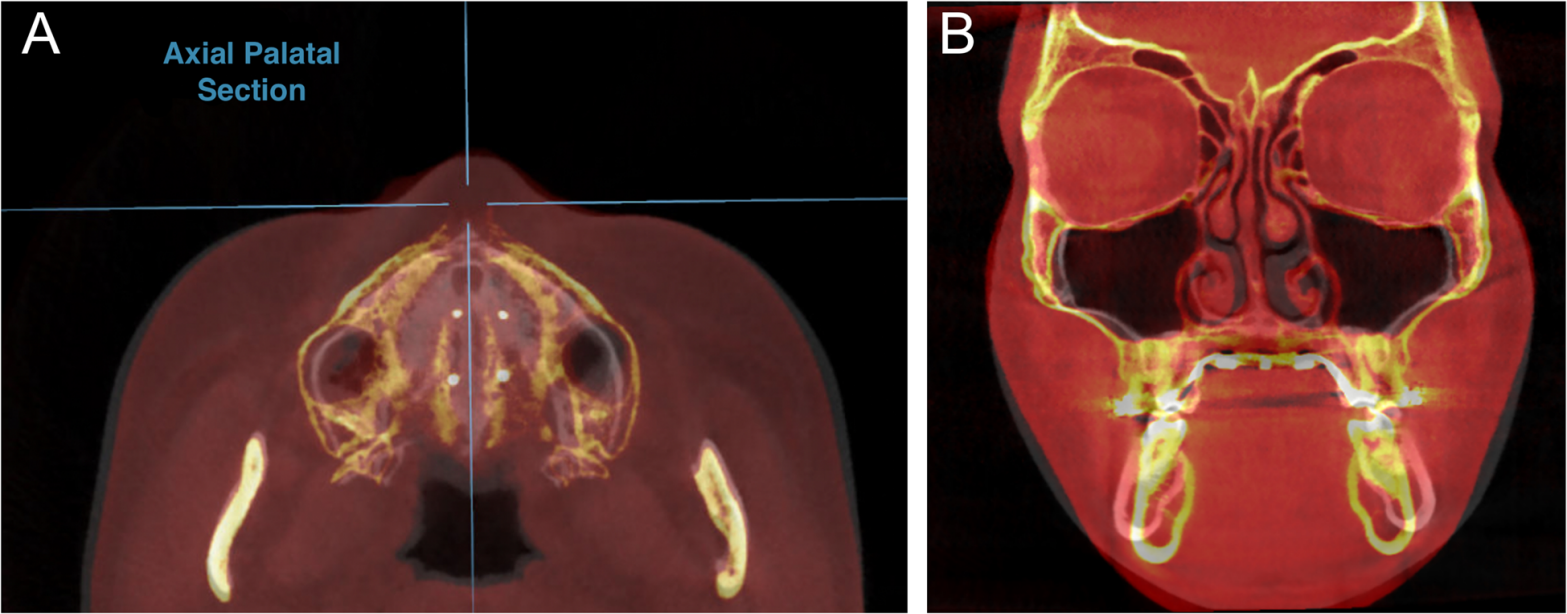
**a** Superimposition of pre- and post-expansion CBCT images. **b** Superimposed image of an MSE patient in the coronal zygomatic section

**Fig. 4:**
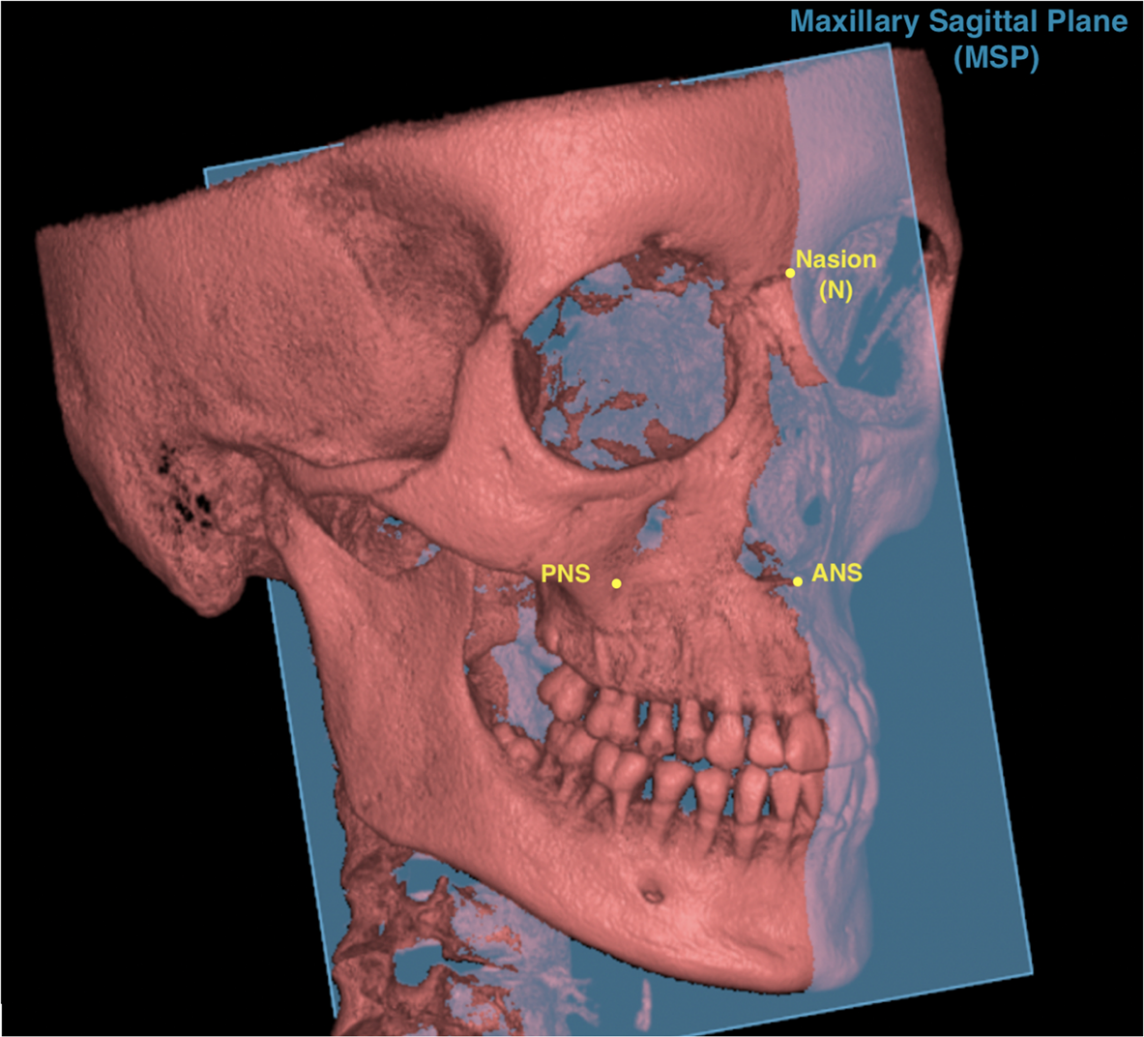
3D demonstration of reference planes. Maxillary sagittal plane (MSP) passing through anterior nasal spine (ANS), posterior nasal spine (PNS), and nasion (N) on the preexpansion CBCT

The MSE device is composed of a jackscrew unit with four parallel holes for micro-implant insertion, with two soft supporting arms on each side which are soldered to the molar bands for stabilizing MSE during the expansion. The jackscrew is seated on the hard palate between the zygomatic buttress bones. The rate of expansion was 0.5–0.8 mm per day until a significant diastema (1–2 mm) was detected. Then the activation rate changed to 0.25 mm per day until the maxillary width was equal or greater than the mandibular width. The MSE appliance remained in place with no further activation for 6 months to allow new bone and suture formation. The first CBCT records were taken before expansion (T0) and the second CBCT were obtained within 3 weeks after completing the expansion (T1) on all patients. All CBCT scans were taken by a NewTom 5G in an 18 × 16 field of view with a 14-bit gray scale. The scan time was 18 s (3.6 s emission time), 110 kV, using an automatic exposure control that modified the milliampere based on the patient’s anatomic density. Data from the CBCT was reconstructed to produce 0.3 mm slices. OnDemand3D (Cybermed, Daejeon, Korea) software was utilized to superimpose CBCT images acquired at T0 and T1, using the anatomical structures of the anterior cranial base [16]. Superimposition method is based on automated processing in matching the voxel gray-scale patterns to prevent human error (Fig. 3). The accuracy of this superimposition method has been successfully investigated in the literature [17]. After superimposing CBCT data of T0 and T1 on Ondemand3D software, reference planes were identified to assess the midpalatal suture opening pattern in the axial palatal section (APS) (Fig. 4).

**Fig. 5:**
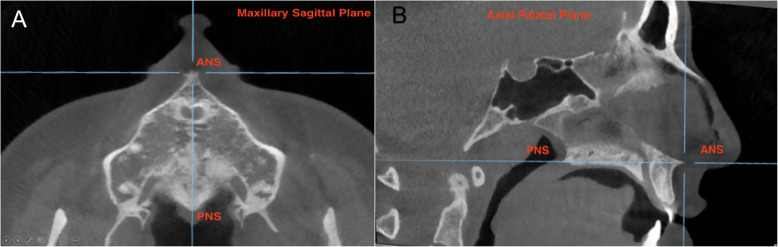
**a** Axial cut shows the maxillary sagittal plane. **b** Axial palatal plane passing through anterior nasal spine (ANS) and posterior nasal spine (PNS). Blue lines represent reference lines

**Fig.6:**
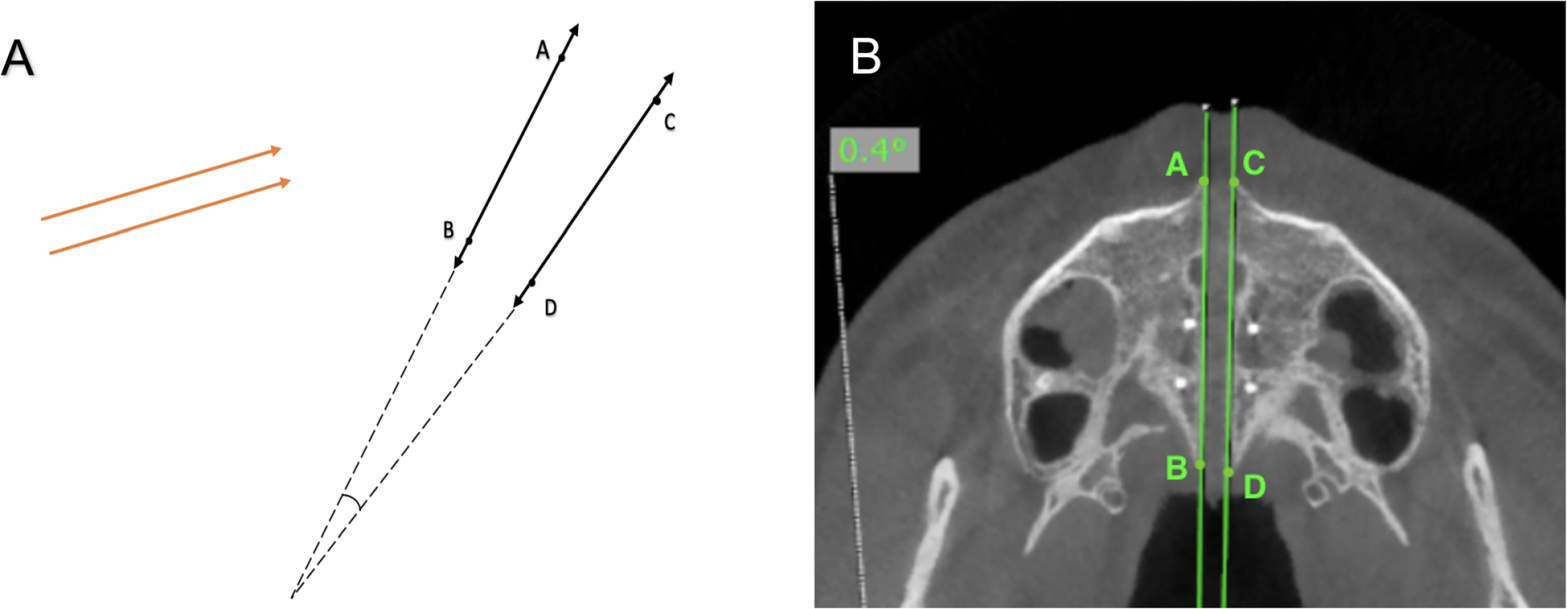
**a** Two parallel lines, in the same plane, form 0°; two non-parallel lines converging at an angle. **b** AB and CD lines are intersecting in a distant point with 0.4°. AB represents right palatal line, and CD represents left palatal line

First, the maxillary sagittal plane (MSP) was determined, passing through anterior nasal spine (ANS), posterior nasal spine (PNS), and nasion (N) on the T0 CBCT images. After this, the axial palatal section (APS) was identified in the axial view (Fig. 5). In the APS, right ANS and right PNS were connected through a straight line (right palatal line), and left ANS and left PNS were also connected through a straight line (left palatal line) (Fig. 6). The angle that is formed by the convergence of right and left palatal lines was measured and called suture opening angle (SOA). If the two conforming lines merged in the posterior region (caused by greater anterior expansion), a positive sign was assigned to the value. In contrast, when the right and left palatal line merged anteriorly (caused by greater posterior expansion), a negative sign was assigned.

Additionally, the pterygopalatine suture opening frequency after expansion was evaluated on the axial cuts of T1.

### Statistical assessment

All statistical analyses were performed using the SPSS version 15.0 software (SPSS Inc., Chicago, IL, USA). A single examiner performed all measurements. To assess method reliability, the same examiner remeasured 25% of randomly selected patient’s degree of palatal opening within a 2-week interval. The examiner conducted measurement without the knowledge of the patients’ names and treatment modality to eliminate any bias. Intraclass correlation coefficient (ICC) test was performed to assess reliability. The T1 value was compared with zero (T0 value), and the *P* value was computed using the Wilcoxon signed-rank test for paired data according to the normality of data distribution. To calculate the reliability of cranium orientation, 25% of the CBCT scans were reoriented based on the landmarks of basion and posterior clinoid process of sella turcica [18] and intraclass correlation coefficient was calculated. The frequency of pterygopalatine suture openings of T0 and T1 was compared between males and females using Fisher’s exact test. The suture opening angle mean between males and females was compared using the Mann-Whitney *U* test.

## Results

**Fig. 7:**
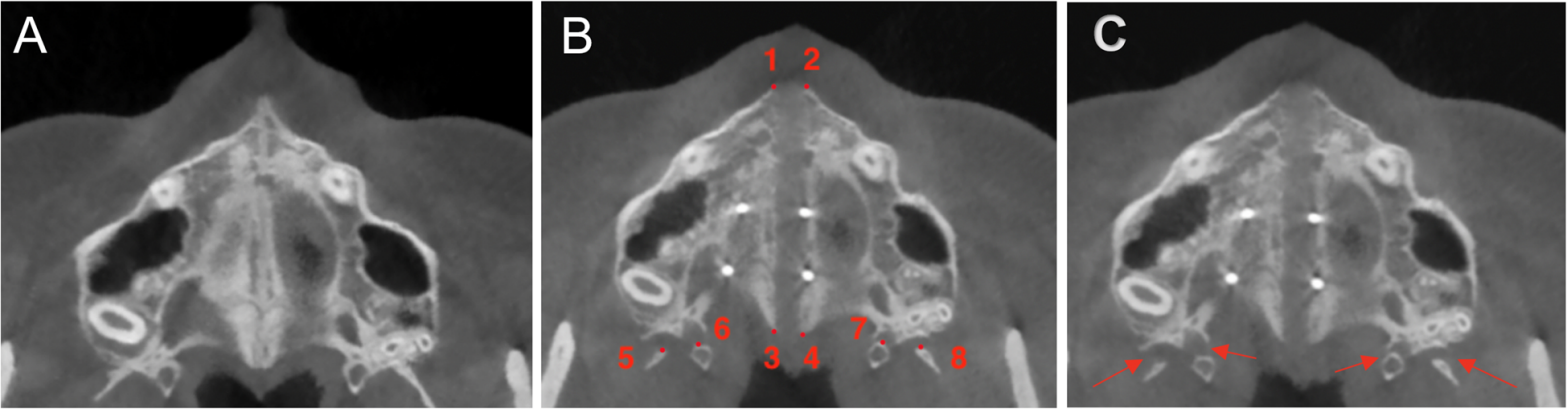
**a** Pre-expansion view on axial palatal section. **b** Post-expansion view on axial palatal section showing anatomical landmarks. 1: right anterior nasal spine (ANS), 2: left anterior nasal spine (ANS), 3: right posterior nasal spine (PNS), 4: left posterior nasal spine (PNS), 5: the most anterior point of lateral plate of the right pterygoid process, 6: the most anterior point of medial plate of the right pterygoid process, 7: the most anterior point of medial plate of the left pterygoid process, and 8: the most anterior point of lateral plate of the left pterygoid process. **c** Disarticulation between medial and lateral pterygoid plates on both right and left pterygopalatine suture after expansion

**Fig. 8:**
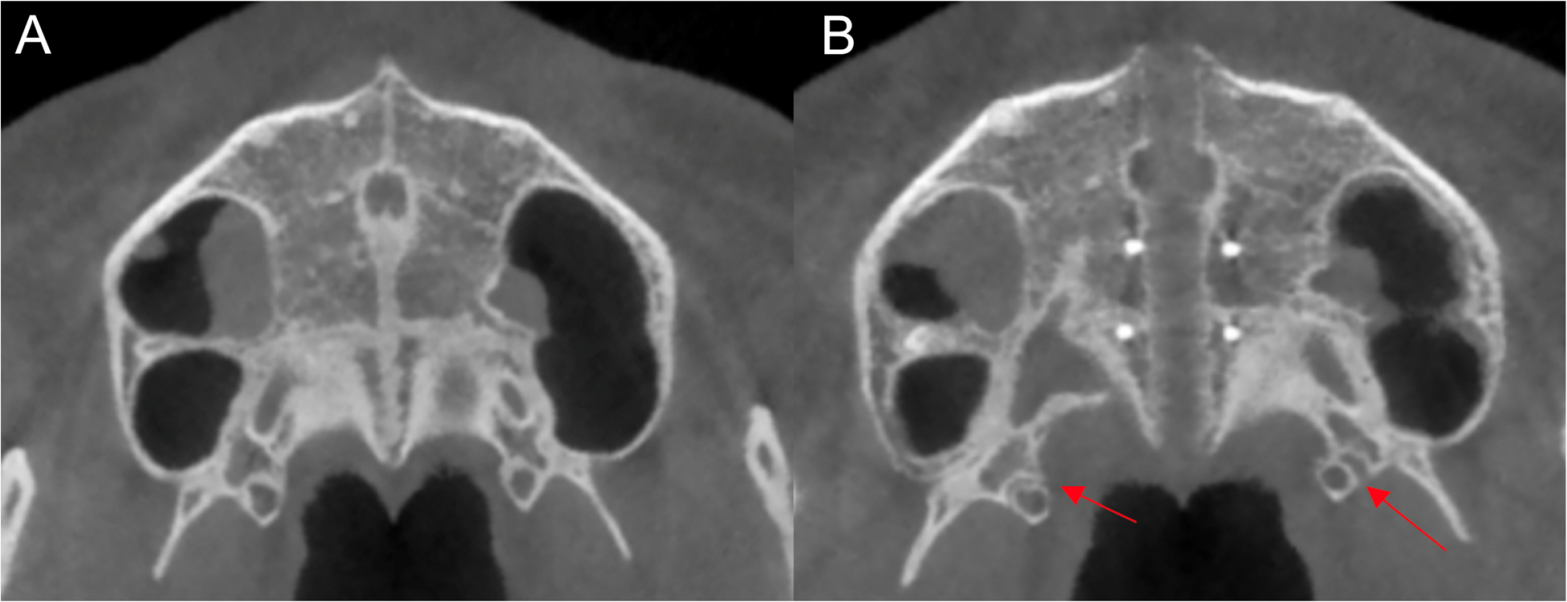
**a** Pre expansion view on axial palatal section. **b** Post-expansion on axial palatal section showing disarticulated medial pterygoid plates on both pterygopalatine sutures

**Fig. 9:**
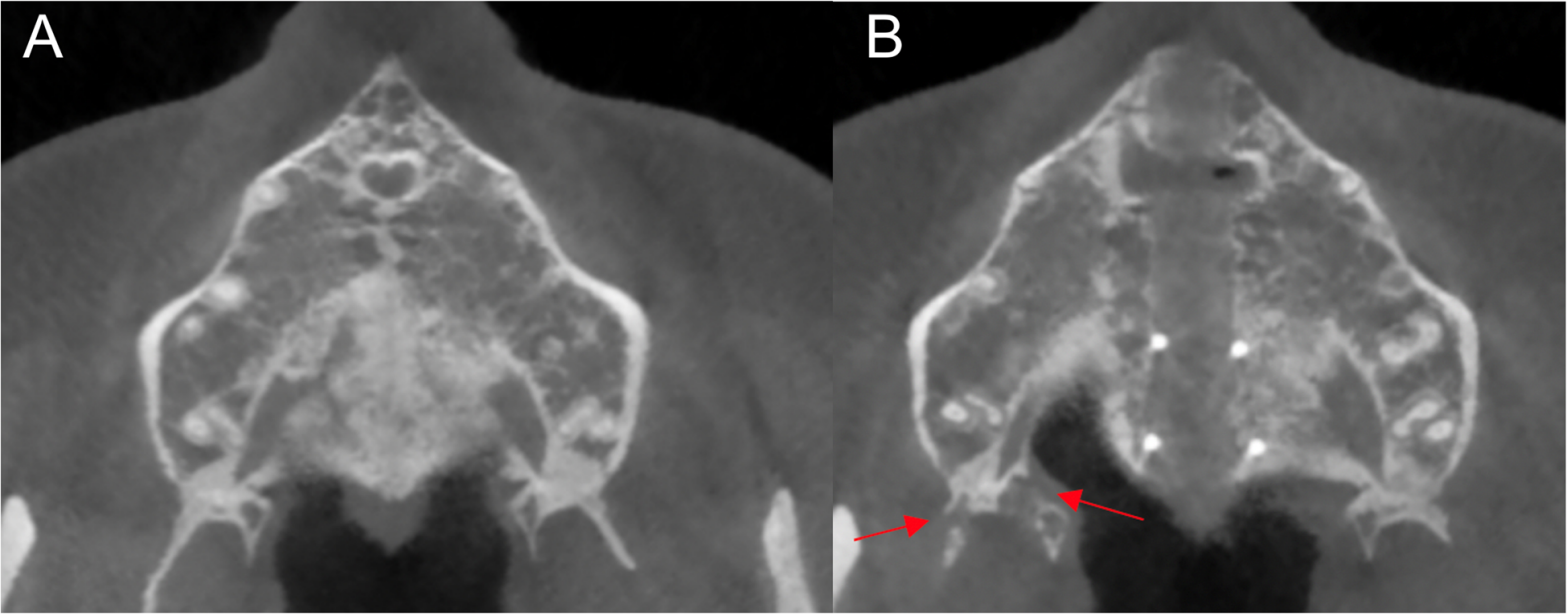
**a** Pre-expansion view on axial palatal section. **b** Post-expansion view on axial palatal section showing the split in medial and lateral pterygoid plates on right pterygopalatine suture

After MSE treatment, the mean suture opening angle (SOA) was 0.57°, ranging from − 0.80° to 1.30° (Table 1). Additionally, three out of fifty (6%) subjects had SOA above 1°. Also, three out of fifty (6%) patients presented a negative SOA value. No significant differences were found between males and females in terms of the SOA mean, as shown in Table 2.With regard to the pterygopalatine suture split, eighty-four sutures out of hundred (84.0%) demonstrated openings between the medial and lateral pterygoid plates on both right and left pterygopalatine sutures (*P* < 0.01) (Table 3, Fig. 7c). Partial split was detected in eight patients (5 females, 3 males). Five patients had split only in the medial pterygoid plates of both pterygopalatine sutures (Fig. 8b), and three patients exhibited disarticulation on the right side only (Table 4, Fig. 9b). No significant differences were found in the frequency of suture opening between males and females (*P* = 1.000).

**Table 1:** Analysis of palatal suture opening pattern. T0, pre-expansion; T1, post-expansion

		T0		T1		Change after treatment		*P* value
	Number	Mean	SD	Mean	SD	Mean	SD	
Males	20	0.00	0.00	0.52°	0.33	0.52°	0.33	< 0.01*
Females	30	0.00	0.00	0.60°	0.32	0.60°	0.32	< 0.0001*
Total	50	0.00	0.00	0.57°	0.32	0.57°	0.32	< 0.0001*

*P < 0.01

**Table 2:** Analysis of palatal suture opening pattern between males and females

	Males		Females		*P* value
	Mean	SD	Mean	SD	
Males	0.52°	0.33	0.60°	0.32	0.558

**Table 3:** Frequency of total split of pterygopalatine suture (PPS) between males and females

Parameter	Males	Females	Total	*P* value
Total split of PPS on Rt and Lt sides	17/20 (85.0%)	25/30 (83.3%)	42/50 (84.0%)	1.000

*RT* right, *LT* left

**Table 4:** Frequency of partial split of pterygopalatine suture (PPS) between males and females

Parameter	Males	Females	*P* value
Split of medial pterygoid plates on Rt and Lt PPS	1/20 (5.0%)	4/30 (13.3%)	0.636
Split of PPS on Rt side only	2/20 (10.0%)	1/30 (3.3%)	0.556

For the considered parameters, the ICC value was 0.95.

## Discussion

Throughout the past decades, the palatal suture opening pattern after RPE has been highly investigated. Although there are many reports on the effects of RPE [4, 13, 19], very few studies exist in the literature about the midpalatal suture opening pattern after treatment with MARPE appliances [11].

2D PA cephalometric analysis as well as intraoral occlusal radiographs are considered valid to evaluate the midpalatal disarticulation pattern. However, superimposition of the anatomical structures causes a limited view of the area and error in measurements. On the other hand, 3D images and novel computational software facilitate much more robust and accurate analysis of the anatomical structure changes, utilizing automated superimposition and precise reference planes. The accuracy of CBCT has been well documented [20]. In the present study, we assessed skeletal changes after MSE treatment using CBCT. The midfacial skeletal expander (MSE) is a particular type of microimplant-assisted maxillary expander (MARPE) which has been described in the literature since 2014 [7, 11, 12, 21–23].

Melsen and Melsen described the role of pterygopalatine suture in preventing posterior expansion, stating that this suture limits the amount of expansion and dictates the pattern of expansion with RPE [24]. Even with MARPE, V-shaped expansion was reported in the previous studies [10]. On the other hand, the MSE expansion created more parallel expansion. Regarding the midpalatal suture, it was observed that the suture opening angle (SOA) was very close to 0° (0.57°), which indicates almost parallel splitting of the suture, despite the fact that suture interdigitation becomes more complex after the adolescent stage of development [3]. With MSE, most of the patients had palatal suture opening angle less than 1°. Only three out of fifty (6%) patients presented with SOA greater than 1° (with the maximum of 1.37°), indicating that great majority of the time MSE expanded the posterior maxilla as much as the anterior region. Three out of fifty (6%) patients had a negative SOA (0.80°) which indicates that the posterior region expanded more, a rare finding with other expanders. This difference could be related to external factors such as the presence of anterior crossbite that hinders the movement of the maxilla, the larger bone mess with sutural interlocking, and the predominant posterior expansion force with MSE.

**Fig. 10:**
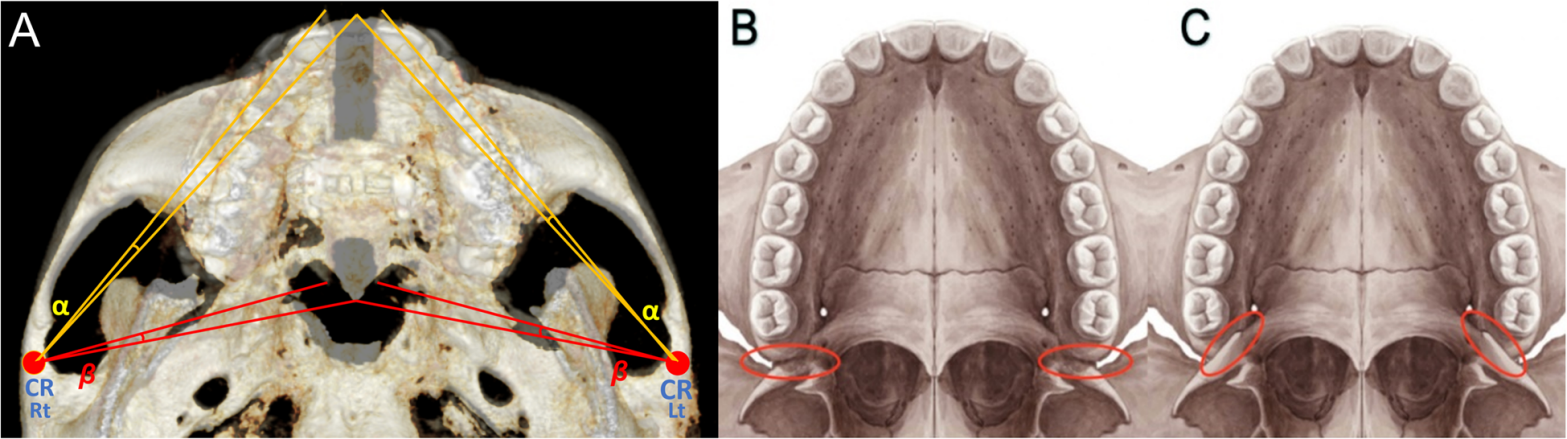
**a** Superimposition of the two images, before and after expansion, illustrating the rotational movement around the center of rotation (CR), near the posterior zygomatic arch. The *α* value represents the degree of rotation between pre- and post-ANS positions. The *β* value represents the degree of rotation between pre- and post-PNS positions. **b** The pterygopalatine suture oriented relatively parallel to maxillary movement. **c** The pterygopalatine suture is not parallel to maxillary movement

The disarticulation or disturbance between the palatine bone and pterygoid process of the sphenoid bone is necessary in order to achieve posterior expansion. Wertz [9] stated that the palatal suture opening created by disarticulation of the maxillary halves was almost always nonparallel, with the wider split being at the anterior region. They demonstrated more space at ANS, progressively narrowing through PNS after RPE. The difference in findings with MSE might be due to the particular design of MSE that promotes posterior expansion force vector as well as the amount of force it can generate. Four micro-implants of MSE are placed with a wide antero-posterior distance between each other, and they are inserted between the zygomatic buttress bones. This posterior position with a wide width between the anterior and posterior implants, which differs from some other appliances, promotes parallel expansion with expansion force directed at the posterior resisting structures. The skeletal impacts of the MSE have been studied and described in the recent years, and it has been successfully utilized in mature patients [25]. Cantarella [26] demonstrated that the zygomaticomaxillary complex shows a rotational movement in the axial plane with its fulcrum near the TMJ area, in contrast to the RPE expansion with fulcrum at the pterygoid process (Fig. 10a). The rotational movement of the midfacial structure produced by MSE (with its fulcrum far away from the maxillary complex) contributes to the relatively parallel opening of midpalatal suture, whereas the RPE (with its fulcrum close to PNS) produces much more V-shaped expansion [9, 27]. The bicortical engagements of implants, by utilizing 11 mm micro-implants, produce also a much greater bony anchorage with a higher stability. This allows adequate expansion force to be used and causes the rotational movement of the midfacial structure by disarticulating pterygopalatine sutures, which in turn produce a relatively parallel expansion of the palatal suture. This posterior skeletal change with MSE can also make a significant contribution to the quality of breathing by reducing the resistance in posterior nasal passage in sleep apnea patients.

Similar to the previous findings, Ramieri [28] showed parallel expansion of the maxilla only after intervention on pterygoid palates. In the current study, 84 sutures out of 100 (84%) presented openings between the medial and lateral pterygoid plates on both right and left sides (*P* < 0.01). Similarly, Cantarella [11] found opening in pterygomaxillary suture as the most common finding in 13 sutures (*N* = 30), along with the partial disengagement detected in 3 sutures after MSE treatment. In the present study, the pattern of partial split in pterygopalatine suture was further analyzed with a larger study group. After observing a symmetrical split on right and left sides of pterygoid processes as the first most common finding (Fig. 7c), the second highest prevalence was the disarticulation only on medial pterygoid plates on right and left sides of pterygomaxillary junctions (Fig. 8b). Lastly, 3 patients out of 50 demonstrated pterygopalatine suture split on the right side only (Fig. 9b).

Different patterns of splitting could be related to the difference in resistance of the suture as well as the bone density and shape of the pterygoid process. Another contributing factor may be the quality of the anchor bone. However, the interdigitation of pterygomaxillary suture is in 3D and complex. And the direction of the suture at the section examined varied with each patient. If the suture direction was parallel with the rotational movement of maxilla, the disarticulation of this suture would not materialize as the opening on axial cuts (Fig. 10b). Small SOAs (− 0.80° to 1.37°) were observed in all cases, which indicates an almost parallel split of midpalatal suture consistently, suggesting the disarticulation of pterygopalatine suture in transverse direction, especially in the cases where the split of the pterygopalatine suture in axial view was not apparent.

Further studies are needed to assess the pterygomaxillary junction in 3D, to clearly understand the possible sliding mechanism of the suture oriented in the direction of maxillary movement during the expansion.

## Conclusion

1. MSE produced a remarkably parallel expansion of midpalatal suture.
2. This study shows that the pterygopalatine suture can be split by MSE appliance without the surgical intervention; the disarticulation of pterygopalatine suture was visible in most of the patients.
3. Further studies in the disarticulation pattern of pterygopalatine suture focusing on transverse sliding mechanism with MSE treatment are desired.

## Data Availability

Data of the present study will not be shared because the same data and materials will be used in further publications where the analysis of different midface bones and sutures will be presented.

## Abbrevıatıons

CBCT: Cone beam computed tomography
MSE: Midfacial skeletal expander
MARPE: Miniscrew-assisted rapid palatal expansion
RPE: Rapid palatal expansion
SARPE: Surgically assisted rapid palatal expansion
SOA: Suture opening angle
IRB: Institutional Review Board
MSP: Maxillary sagittal plane
ANS: Anterior nasal spine
PNS: Posterior nasal spine
N: Nasion
PA: Postero-anterioret
PPS: Pterygopalatine suture al

## References

[1] McNamara JA (2000). Maxillary transverse deficiency. Am J Orthod Dentofacial Orthop..

[2] Haas AJ (1965). The treatment of maxillary deficiency by opening the midpalatal suture. Angle Orthod..

[3] Melsen B (1975). Palatal growth studied on human autopsy material. A histologic microradiographic study. Am J Orthod..

[4] Gurel HG, Memili B, Erkan M, Sukurica Y (2010). Long-term effects of rapid maxillary expansion followed by fixed appliances. Angle Orthod..

[5] Lin L, Ahn HW, Kim SJ, Moon SC, Kim SH, Nelson G (2015). Tooth-borne vs bone-borne rapid maxillary expanders in late adolescence. Angle Orthod..

[6] Williams BJ, Currimbhoy S, Silva A, O'Ryan FS (2012). Complications following surgically assisted rapid palatal expansion: a retrospective cohort study. J Oral Maxillofac Surg..

[7] Lee RJ, Moon W, Hong C (2017). Effects of monocortical and bicortical mini-implant anchorage on bone-borne palatal expansion using finite element analysis. Am J Orthod Dentofacial Orthop..

[8] Wilmes B, Ludwig B, Vasudavan S, Nienkemper M, Drescher D (2016). The T-Zone: Median vs. Paramedian insertion of palatal mini-implants. J Clin Orthod..

[9] Wertz RA (1970). Skeletal and dental changes accompanying rapid midpalatal suture opening. Am J Orthod..

[10] Wilmes B, Nienkemper M, Drescher D (2010). Application and effectiveness of a mini-implant- and tooth-borne rapid palatal expansion device: the hybrid hyrax. World J Orthod..

[11] Cantarella D, Dominguez-Mompell R, Mallya SM, Moschik C, Pan HC, Miller J (2017). Changes in the midpalatal and pterygopalatine sutures induced by micro-implant-supported skeletal expander, analyzed with a novel 3D method based on CBCT imaging. Prog Orthod..

[12] Song KT, Park JH, Moon W, Chae JM, Kang KH (2019). Three-dimensional changes of the zygomaticomaxillary complex after mini-implant assisted rapid maxillary expansion. Am J Orthod Dentofacial Orthop..

[13] Lione R, Ballanti F, Franchi L, Baccetti T, Cozza P (2008). Treatment and posttreatment skeletal effects of rapid maxillary expansion studied with low-dose computed tomography in growing subjects. Am J Orthod Dentofacial Orthop..

[14] Christie KF, Boucher N, Chung CH (2010). Effects of bonded rapid palatal expansion on the transverse dimensions of the maxilla: a cone-beam computed tomography study. Am J Orthod Dentofacial Orthop..

[15] Andrews LFAW (2000). The six elements of orofacial harmony. Andrews J Orthod Orofac Harmony..

[16] Cevidanes LH, Heymann G, Cornelis MA, DeClerck HJ, Tulloch JF (2009). Superimposition of 3-dimensional cone-beam computed tomography models of growing patients. Am J Orthod Dentofacial Orthop..

[17] Weissheimer A, Menezes LM, Koerich L, Pham J, Cevidanes LH (2015). Fast three-dimensional superimposition of cone beam computed tomography for orthopaedics and orthognathic surgery evaluation. Int J Oral Maxillofac Surg..

[18] Woller JL, Kim KB, Behrents RG, Buschang PH (2014). An assessment of the maxilla after rapid maxillary expansion using cone beam computed tomography in growing children. Dental Press J Orthod..

[19] Doruk C, Bicakci AA, Basciftci FA, Agar U, Babacan H (2004). A comparison of the effects of rapid maxillary expansion and fan-type rapid maxillary expansion on dentofacial structures. Angle Orthod..

[20] Mah JK, Danforth RA, Bumann A, Hatcher D (2003). Radiation absorbed in maxillofacial imaging with a new dental computed tomography device. Oral Surg Oral Med Oral Pathol Oral Radiol Endod..

[21] Cantarella D, Dominguez-Mompell R, Moschik C, Mallya SM, Pan HC, Alkahtani MR (2018). Midfacial changes in the coronal plane induced by microimplant-supported skeletal expander, studied with cone-beam computed tomography images. Am J Orthod Dentofacial Orthop..

[22] Garcez AS, Suzuki SS, Storto CJ, Cusmanich KG, Elkenawy I, Moon W (2019). Effects of maxillary skeletal expansion on respiratory function and sport performance in a para-athlete - a case report. Phys Ther Sport..

[23] Abedini S, Elkenawy I, Kim E, Moon W (2018). Three-dimensional soft tissue analysis of the face following micro-implant-supported maxillary skeletal expansion. Prog Orthod..

[24] Melsen B, Melsen F (1982). The postnatal development of the palatomaxillary region studied on human autopsy material. Am J Orthod..

[25] Carlson C, Sung J, McComb RW, Machado AW, Moon W (2016). Microimplant-assisted rapid palatal expansion appliance to orthopedically correct transverse maxillary deficiency in an adult. Am J Orthod Dentofacial Orthop..

[26] Cantarella D, Dominguez-Mompell R, Moschik C, Sfogliano L, Elkenawy I, Pan HC (2018). Zygomaticomaxillary modifications in the horizontal plane induced by micro-implant-supported skeletal expander, analyzed with CBCT images. Prog Orthod..

[27] Gopalakrishnan U, Sridhar P (2017). Assessment of the dental and skeletal effects of fan-type rapid maxillary expansion screw and Hyrax screw on craniofacial structures. Contemp Clin Dent..

[28] Ramieri GA, Spada MC, Austa M, Bianchi SD, Berrone S (2005). Transverse maxillary distraction with a bone-anchored appliance: dento-periodontal effects and clinical and radiological results. Int J Oral Maxillofac Surg..

